# Where do the elderly die? The impact of nursing home utilisation on the place of death. Observations from a mortality cohort study in Flanders

**DOI:** 10.1186/1471-2458-6-178

**Published:** 2006-07-06

**Authors:** Gilberte Van Rensbergen, Tim S Nawrot, Ettiene Van Hecke, Benoit Nemery

**Affiliations:** 1 Department of Public Health, Katholieke Universiteit Leuven, Leuven, Belgium; 2 Instituut voor Sociale en Economische Geografie, Katholieke Universiteit Leuven, Leuven, Belgium

## Abstract

**Background:**

Most of the research concerning place of death focuses on terminally ill patients (cancer patients) while the determinants of place of death of the elderly of the general population are not intensively studied. Studies showed the influence of gender, age, social-economical status and living arrangements on the place of death, but a facet not taken into account so far is the influence of the availability of nursing homes.

**Methods:**

We conducted a survey of deaths, between January 1999 and December 2000 in a small densely populated area in Belgium, with a high availability of nursing homes (within 5 to 10 km of the place of residence of every elderly). We determined the incidence of total mortality (of subjects >60 years) from local official death registers that we consulted via the priest or the mortician of the local parish, to ask where the decedent had died and whether the deceased had lived in a nursing home. We compared the distribution of the places of death between parishes with a nursing home and with parishes without nursing home.

**Results:**

240 women and 217 men died during the two years study period. Only 22% died at home, while the majority (78%) died in an institutional setting, either a hospital (50%) or a nursing home (28%). Place of death was influenced by individual factors (age and gender) and the availability of a nursing home in the 'own' parish. The chance of in-hospital death was 65% higher for men (95% Confidence Interval [CI]: 14 to 138%; p = 0.008) and decreased by 4% (CI: -5.1% to -2.5%; p < 0.0001) for each year increase in age. Independent of gender and age, the chance of in-hospital death was 41% (CI: -60% to -13%; p = 0.008) lower in locations with a nursing home.

**Conclusion:**

Demographic, but especially social-contextual factors determine where elderly will end their life. The majority of elderly in Flanders die in an institution. Age, gender and living situation are predictors of the place of death but the embedment of a nursing home in the local community seems to be a key predictor.

## Background

We investigated if geographical variations in nursing home utilisation are due to differences in local nursing home availability and what the influence of this is on the place of death. Optimal quality of life is influenced by various factors, such as physical, psychological, and social well being [1]. Quality of life is also relevant to for the place where elderly spend the last period of their lives.

Nursing homes and hospitals increasingly have become the most common site of death [2] in Western Europe [2,3]. Belgian data on the place of death and his determinants are lacking. Since the early 1990s, studies in other countries have explored the determinants of the place of death in specific segments of the population [3-11] especially among end-of-life care of patients with cancer in relation to dying at home. Many studies do not focus on all places of death (home, nursing home and hospital) but compare dying at home with one of the other possibilities and this often in association with cost-savings at the end of life.

While in general most people die in an institutional setting, population surveys indicate that more than 70% of people would prefer to die at home which is less costly [12]. However, dying at home might not always be the best place for everyone, as this option depends on the individual pathological situation (e.g. the need of therapy) and having someone who takes care (husband, wife or children). If death at home is not an option, nursing home death is more in accord with patients' preferences, due to human reasons (home replacing environment).

The place of death is a multifactorial phenomenon [8-15]. Studies have clearly shown the influence of gender, age, socioeconomic factors and marriage status (living arrangements) on the place of death, showing that more women and persons living alone die in a nursing home [4-10].

However, a facet not taken into account so far, is the influence of the availability of nursing homes on the pattern of the place of death [12,13] The availability of nursing homes in Flanders (Northern Belgium) is very high and in principle there is access to all who want it: no distance, no social-economic thresholds, no health limitations [13]. Moreover, there is a merged system of rest home and nursing home, which means that the elderly can move between different levels of care without leaving the facility (no transfers) [13,14]. In other words there is access for healthy, semi-healthy and disabled elderly. Due to the fundamental importance of quality dimensions in nursing homes and the increasing concern of the government, there should not be a difference of quality of death between hospital and nursing home in Belgium (if hospitalisation is not medical and ethical necessary). They only differ in their cost implications [14].

We investigated, in a densely populated area in Northern Belgium, whether variation in nursing home utilisation is explained by 1/differences in individual characteristics (such as gender, age, marital status), and 2/by the distribution of local nursing homes.

## Methods

**Figure 1:**
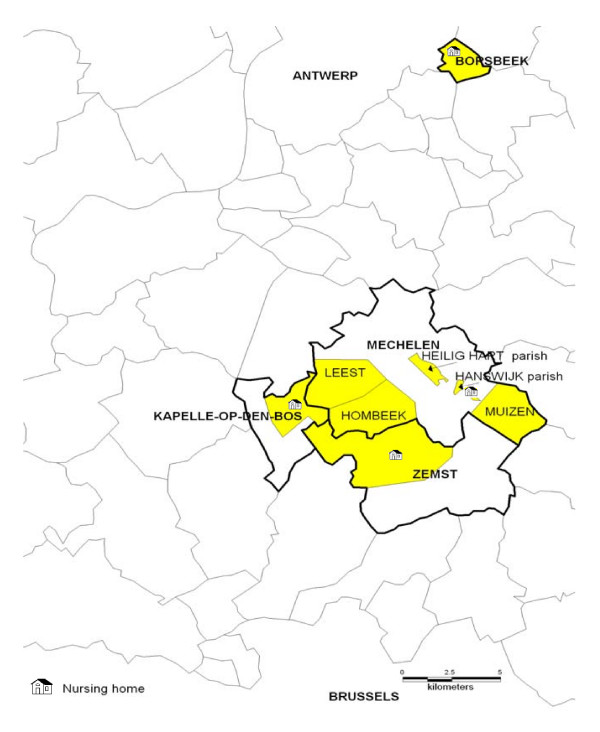
**Map of the study area**. The (rural) parishes with and without nursing home border. Borsbeek is added as control, just as the 2 urban parishes of Mechelen. The parish borders are indicated. There is a difference of 15% in nursing home utilisation between parishes with or without nursing home (44% versus 29%; CI 5.7 to 24.1; p = 0.0008).

We conducted a survey of deaths, between January 1999 and December 2000 in a small area in Belgium. In a geographically defined area around Mechelen (figure 1), in a convenience sample of four parishes with a nursing home (Borsbeek, Kapelle-op-den-Bos, Mechelen Hanswijk, Zemst) and four parishes without a nursing home (Hombeek, Leest, Mechelen Heilig Hart, Muizen), we determined the incidence of total mortality (of subjects >60 years) from local official death registers that we consulted via the priest of the parish. The parish registers of the Netherlands (existing since the Council of Trente in 1545) are internationally considered as reliable research sources by historians. In the parishes under study the dead registers contain all the deceased persons. Deaths which occurred outside the parish (e.g. patients who died in a nursing home or hospital or elsewhere outside the parish) were also covered, because residents of nursing homes or hospitals are normally buried in the parish where they come from (the code of Napoleon). In one parish the priest refused to take part in the study and the funeral register of the mortician was consulted. The registers of the parish include the name of the deceased person, place and date of birth, place and date of death, and information on the marital status (except the urban parish of Mechelen Hanswijk). In the urban parish of Mechelen Heilig Hart, the specific place of death (hospital or nursing home) could not be traced for 8 persons. The study was performed in accordance with the Helsinki Declaration an d approved by the Ethics Committee of the University of Leuven.

We used chisquare statistic and multiple logistic regression analysis to estimate the influence of the availability of a nursing home in the parish on the distribution of the place of death. In multiple logisitic regression we adjusted our associations for age, gender and marital status (SAS software version 8.1).

## Results

Of the 457 subjects the place of death was not retrieved for 8 subjects. The remaining 449 elderly, 213 women and 236 men, who died during the two years study period in the 8 studied parishes, had a mean age of 83.4 (SD: 8.6) and 78.2 (9.0) years (p < 0.0001), respectively; 44.9% had a co-resident (was married or lived together). 25% died suddenly.

Across the parishes there were no significant differences with regard to the gender distribution, age, and living situation of the deceased, while the distribution of the place of death differed across the parishes with or without a nursing home (table 1 and 2). 22% died at home (this includes 2 who died elsewhere, such as public road), while the majority (78%) died in an institutional setting, either a hospital (50%) or a nursing home (28%). Only 14% of those who died in the hospital came from a nursing home.

**Figure 2:**
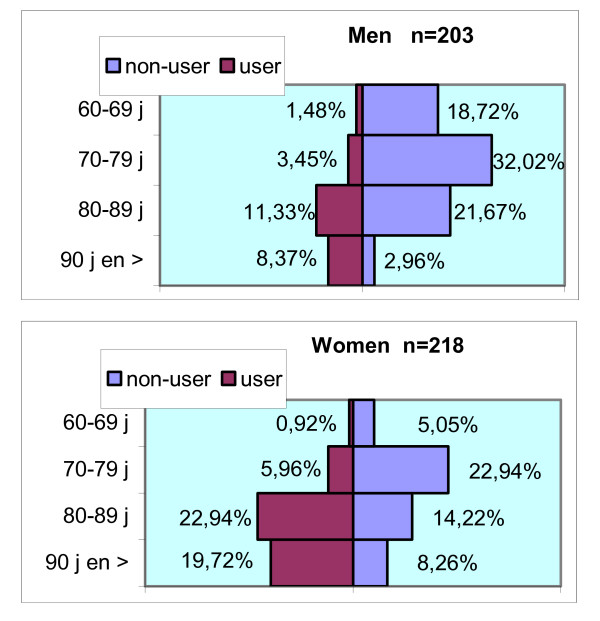
Age distribution for deceased men and women, with or without the use of a nursing home in their life.

Place of death was influenced by individual factors such as gender, age and living situation as well as the presence of a nursing home located in the parish. Across the age distribution of our sample more women than men (50% *vs *25%; p < 0.0001) resided in a nursing home during the last period (months or years) of their life (figure 2). Those who lived alone were 7.76 (p < 0.0001) times more likely to go to a nursing home. The chance of in-hospital death was 65% higher for men (95% Confidence Interval [CI]: 14 to 138%; p = 0.008) and decreased by 4% (CI: -5.1% to -2.5%; p < 0.0001) for each year increase in age. The chance for dying in a nursing home was 62.6% lower for men (CI: 38.3 to 77.3%; p < 0.0001) and increased by 11% (CI: 7.5 to 14.7; p < 0.0001) for each year increase in age. While the chance of dying at home was not influenced by gender (p = 0.26) it does decrease by 3.8% for each year increase in age (p = 0.003).

**Figure 3:**
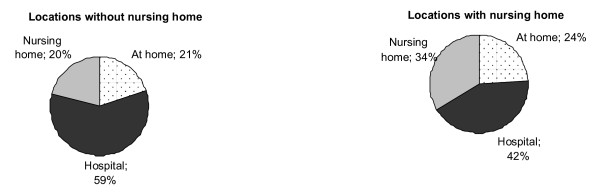
Distribution of the place of death for locations with and without a nursing home.

Both before (table 2 and figure 3) and after adjustment for gender and age the chance of in-hospital death was 41% (CI: -60% to -13%; p = 0.008) lower in locations with a nursing home. Additional adjustment for 'marital' status did not alter these associations.

**Figure 4:**
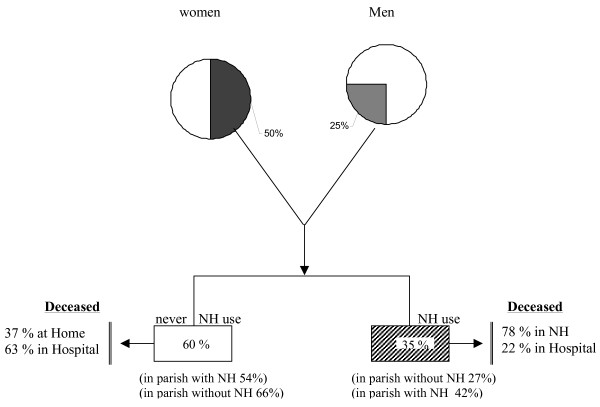
**Place of death of the 60 plus population**. In the parishes under study the death registers contain all the deceased persons. Deaths which occurred outside the parish (e.g. patients who died in a nursing home (NH) or hospital outside the parish) were also covered. Residents of nursing homes or hospitals are registered in the parish register where they com from.

10% of the men and 19% of the women who died in a hospital had resided in a nursing home before. Of the subjects who never resided in a nursing home (60%), 37% died at home and 63% in the hospital (Figure 4).

## Discussion

Recent public attention has focused on quality of care for the dying. [6] Where one dies is an important individual and public health concern. Place of death is influenced by individual characteristics and by the distribution of 'local' health provisions. No national study in Belgium has adequately characterized the place of death in relation to subject characteristics. European data on the influence of local health care provisions on the place of death are lacking. This study showed that both individual and environmental-related factors, such as the availability of a nursing home in the 'own' parish, determine the place of death in the elderly. In line with observations from others, [3,12,13] women were more likely to die in a nursing home than were men.

Independently of gender and age the chance of dying in a hospital was 41% lower in locations with a nursing home. These results must be interpreted within the context of the characteristics of the study area. Flanders is one of the most densely populated areas in Europe, with a population density of 434 inhabitants per squared km [15]. In our study, the parishes without a nursing home border to parishes with a nursing home, which means that a nursing home was never further than 5 to10 km of the place of residence of every elderly.

Therefore, our results suggest that social-contextual factors, such as the embedment of a nursing home in the local community, determine the place of death of the elderly. Embedment can be defined as being part of the social network of the parish.

With the "baby boom generation" starting to reach retirement, reforming the elderly care with regard to the place of death, will probably require an increase of all sorts of local alternative resources availability rather than increasing the capacity of the existing nursing homes. We assume that promoting and expanding of the domestic help, informal carer support, home care and mainly palliative home care services, implemented in small geographical entities, will lead to an increase in the possibility to die at home.

We noted an inverse association between the chance of in-hospital death and age. Those dying at older age were the least likely to die in a hospital but were more likely to die in a nursing home. Much of the discussion about care of the dying focuses on deaths from cancer [9-11,17]. In a survey on oncological services in the district of Antwerp, 77% of the cancer patients died in hospital, 3% in a nursing home and 20% at home [16]. A comparable distribution of place of death in the general population as in our study was observed in 2001 in the German speaking part of Switzerland, where death occurred most frequently in hospital (37.2%) followed by a nursing home (33.5%) and dying at home (22.7%) [17].

Our study must be interpreted with its limitations including the relative small sample and the use of parish registers. However, based on social and demographic records we may assume that, in these places and at the moment of the study, more than 99% of the inhabitants had a religion burial and consequently registered in the parish registers [15]. Therefore, we strongly believe that these registers are representative for the total population at the geographical level of our study. Even people who moved out the parish to go to a nursing home or hospital outside the parish are noted in the parish registries (Napoleon code). In our study, the use of national mortality data was not an option since our aim was to study the influence of locally embedded social structures on the place of death and these registries do not provide not enough detailed information at the locally level required to address this research question. The present results are also limited because it was not possible to include in the analysis detailed information on the acuity of the illness, since the cause of death was often unknown. However, it is unlikely that there are differences across the parishes with regard to the acuity of the illness.

There is no norm where people should die. This depends on the acuity of the illness as well as personal preferences, the ability and willingness of the co-resident and the available possibilities of formal home care. The results of the present study provide an important insight into the transitions in care in the last period of life, showing that independently of the known determinants (age, gender, living situation) the place of death is triggered by care provisions at a very local level.

## Conclusion

Demographic but also social-contextual factors determine where elderly will end their life. Age, gender and living situation are predictors of the place of death but the embedment of a nursing home in the local community seems to be a key predictor. Even in an area with an availability of nursing homes within 5 to 10 km of the place of residence, the chance of in-hospital death is inversely associated with a nursing home being located in the 'own' parish of the resident.

## Competing interests

The author(s) declare that they have no competing interests.

## Authors' contributions

GVR designed the study, obtained data, and constructed the database and wrote the first draft of the manuscript. TN did the statistical analysis. EVH produced the map. BN and TN participated in various stages and prepared the final draft of the manuscript.

## Pre-publication history

The pre-publication history for this paper can be accessed here:



## Figures and Tables

**Table 1:** Characteristics of deceased persons in 8 locations

**Parishes**	**Number of deaths**	**Men (%)**	**Age at death (SD)**	**Living situation (m + w) **Living alone (%)
**Without Nursing home**				
Hombeek	82	45 (55)	80.3 (9.4)	46 (56)
Leest	32	14 (44)	79.6 (8.1)	14 (44)
Muizen	50	21 (42)	79.7 (7.2)	18 (45) 10 unknown
Mechelen H. Hart	40	24 (60)	79.9 (8.7)	23 (58)
**Subtotal**	**204**	**104 (51)**	**79.9 (8.6)**	**101 (52)**
				
**With Nursing home**				
Borsbeek	79	40 (51)	81.4 (9.6)	38 (48)
Kapelle-O/D Bos	33	14 (42)	80.5 (9.3)	21 (64)
Zemst	75	32 (43)	81.0 (9.5)	50 (67)
Mechelen Hanswijk	66	27 (41)	84.0 (9.4)	unknown
**Subtotal**	**253**	**113 (45)**	**82.5 (9.5)**	**109 (58)**
				
**TOTAL**	**457**	**217 (47)**	**81.0 (9.0)**	**210 (55)**

Values are numbers (%), or mean (SD)

Missing values are not taken into account (66 + 10 unknown).

**Table 2:** Distribution of place of death in parishes with or without nursing home

**Parishes**	**Place of death**			**Deceased who**	
	At Home (%)	Nursing home (%)	Hospital (%)	never used a nursing home	used a nursing home (%)
**Without Nursing home**					
Hombeek	18 (22)	20 (24)	44 (54)	55 (67)	26 (32)
Leest	9 (28)	2 (6)	21 (66)	29 (91)	3 (9)
Muizen	11(22)	11 (22)	28 (56)	30 (60)	15 (30)
Mechelen H. Hart *	2(6)	7 (22)	23 (72)	21 (53)	11 (28)
**Subtotal**	**40 (20)**	**40 (20)**	**116 (59)**	**135 (66)**	**55 (27)**
					
**With Nursing home**					
Borsbeek	25 (31)	20 (25)	34 (43)	38 (48)	32 (41)
Kapelle-O/D Bos	10 (30)	8 (24)	15 (46)	23 (70)	10 (30)
Zemst	19 (25)	22 (29)	34 (45)	46 (61)	29 (39)
Mechelen Hanswijk	6 (9)	36 (55)	24 (36)	30 (45)	36 (55)
**Subtotal**	**60 (24)**	**86 (34)**	**107 (42)**	**137 (54)**	**107 (42)**
					
**TOTAL**	**100 (22)**	**126 (28)**	**223 (50)**	**272 (60)**	**162 (35)**

Distribution of place of death between places with and without nursing home (chisquare: p = 0.0008)

*place of death was not known for 8 subjects. (n = 449)

From 15 in-hospital deaths the use of a nursing home could not be traced. (n = 434)

Mechelen H. Hart and Mechelen Hanswijk are urban parishes; the others are rural.
